# Plant image identification application demonstrates high accuracy in Northern Europe

**DOI:** 10.1093/aobpla/plab050

**Published:** 2021-07-27

**Authors:** Jaak Pärtel, Meelis Pärtel, Jana Wäldchen

**Affiliations:** 1 Hugo Treffner Gymnasium, Munga 12, Tartu 51007, Estonia; 2 Institute of Ecology and Earth Sciences, University of Tartu, Lai 40, Tartu 51005, Estonia; 3 Max Planck Institute for Biogeochemistry, 07745 Jena, Germany

**Keywords:** Artificial intelligence, automated plant species identification, citizen science, convolutional neural networks, deep learning, Estonian flora, Flora Incognita, identification application, plant identification

## Abstract

Automated image-based plant identification has experienced rapid development and has been already used in research and nature management. However, there is a need for extensive studies on how accurately automatic plant identification works and which characteristics of observations and study species influence the results. We investigated the accuracy of the *Flora Incognita* application, a research-based tool for automated plant image identification. Our study was conducted in Estonia, Northern Europe. Photos originated from the Estonian national curated biodiversity observations database, originally without the intention to use them for automated identification (1496 photos, 542 species) were examined. *Flora Incognita* was also directly tested in field conditions in various habitats, taking images of plant organs as guided by the application (998 observations, 1703 photos, 280 species). Identification accuracy was compared among species characteristics: plant family, growth forms and life forms, habitat type and regional frequency. We also analysed image characteristics (plant organs, background, number of species in focus), and the number of training images that were available for particular species to develop the automated identification algorithm. From database images 79.6 % of species were correctly identified by *Flora Incognita*; in the field conditions species identification accuracy reached 85.3 %. Overall, the correct genus was found for 89 % and the correct plant family for 95 % of the species. Accuracy varied among different plant families, life forms and growth forms. Rare and common species and species from different habitats were identified with equal accuracy. Images with reproductive organs or with only the target species in focus were identified with greater success. The number of training images per species was positively correlated with the identification success. Even though a high accuracy has been already achieved for *Flora Incognita*, allowing its usage for research and practices, our results can guide further improvements of this application and automated plant identification in general.

## Teaser

During the age of a global biodiversity crisis, it is increasingly important to recognize which plant species surround us in order to protect them. One solution to improve knowledge about plants is using image-based identification applications powered by artificial intelligence. We examined several thousand images of hundreds of species from Northern Europe to explore one such application, *Flora Incognita*. We found a high accuracy but not in all plant groups. Performance of the application was also dependent how many training images machine learning had used per species. Even more accurate identification could be expected with additional training data.

## Introduction

Knowledge about biodiversity is critical for nature conservation. In the age of consistent global loss of species and habitats (Ceballos *et al.* 2015), the need for trained experts with good species knowledge is of growing importance to enforce the necessary protection measures for the flora and vegetation. Unfortunately, the species knowledge has recently been receding amongst the public (Hopkins and Freckleton 2002). Plant blindness—an individual’s inability to notice plants around them and appreciate their importance—has increased, especially among the youth (Jose *et al.* 2019). While artificial intelligence such as readily available mobile device applications potentially makes plant identification more widely available, there is a need to test their promises and limitations, especially before using them in research and nature management.

Technical developments have gradually found their way into plant identification (Joly *et al.* 2014; Goëau *et al.* 2016; Lee *et al.* 2015; Wäldchen and Mäder 2018; Christin *et al.* 2019). This is the result of the enormous achievements in the field of machine learning. The combination of increasing computer power and the recent boost in data availability led to significant advances in machine learning algorithms, notably deep learning technologies. From different deep learning methods, convolutional neural networks (CNNs) (LeCun *et al.* 2015) allow the applications to have superior recognition performance (Krizhevsky *et al.* 2017; Russakovsky *et al.* 2015) and therefore form the basis of successful and efficient automated plant identification (Wäldchen and Mäder 2018; Christin *et al.* 2019). Deep CNNs have shown accuracies equivalent to human performance on general object recognition tasks (Russakovsky *et al.* 2015) and on fine-grained species identification tasks (Bonnet *et al.* 2018; Goëau *et al.* 2018; Valan *et al.* 2019). How deep learning has improved classification accuracy in plant identification is demonstrated in the results of the PlantCLEF challenges, a plant identification competition hosted since 2011 as an international evaluation forum (http://www.imageclef.org/). Identification performance improved year after year despite the task becoming more complex by increasing the number of plant species. A tremendous gain in classification accuracy is visible in 2015 when the identification accuracy increased from 45 to 65 % while the species correctly identified doubled from 500 to 1000 species. This improvement is attributed to the adoption of deep learning CNNs (Affouard *et al.* 2017).

These approaches resulted recently in usable tools for automated plant identification via mobile devices. Prominent examples here are *Pl@ntNet* (Goëau *et al.* 2014), *iNaturalist* (iNaturalist 2021) and *Flora Incognita* (Mäder *et al.* 2021). All three are developed within a scientific context and already have a high popularity with several million downloads and could be a future way of accelerating the process of learning the species and collecting data about their distribution and dynamics (Bonnet *et al.* 2020). In the current study we focus on *Flora Incognita*, which is a widely used application for automated image-based plant identification in Europe.

Automated image identification can be complemented by additional data, for example observation metadata such as location or time of the year. *Pl@ntNet* users can select a project from different regions, each project containing plants from a certain region (e.g. plants of Western Europe, North Africa etc.). *iNaturalist* and *Flora Incognita* give the seasonality of the observations for the taxon, making it easier for the user to select the correct species. Applications provide several images and website links to the suggested taxa, from which the users can check the identification validity. While *Pl@ntNet* and *iNaturalist* are evaluating the automatic recognition collaboratively by the user community, *Flora Incognita* has not yet integrated this mechanism. However, with the large number of observations, it will be more and more difficult in the future to evaluate each observation by humans.

For wider use (e.g. in citizen science projects or for plant species monitoring; Mahecha *et al.* 2021), it is critical to determine the applications’ accuracy. So far, plant image identification algorithms have mainly been tested and compared on different benchmark data sets (e.g. in the PlantCLEF challenge) but there have been just a few attempts to evaluate the applications’ performance under realistic use conditions. So far those have been staged in a laboratory environment (Lüdemann 2020); with pictures taken from a database (Biluk *et al.* 2020; Jones 2020) or have used a limited number of field observations (Schmidt and Steinecke 2019). Still, there is a lack of studies which evaluate artificial intelligence-based plant identification using a large number of both database and field observations comparatively and explore identification success among taxonomic and ecological groups. The possible impact of image characteristics (e.g. background, number of species in focus) would also be very valuable information to obtain a higher identification success.

The aim of this study is to determine the identification accuracy of *Flora Incognita* for the Estonian flora. We compared *Flora incognita*’s ability to identify plants from pictures taken from the Estonian curated biodiversity observations database and from field observations taken with the application. Furthermore, we examined the application’s performance across larger plant families, growth forms, Raunkiær’s life-form categories, species’ main habitat types and frequency in Estonia. We explored the importance of image characteristics (reproductive or vegetative organs, one or more species in the image, and background). Finally, we tested if the amount of training images per species is related to identification success.

## Materials and Methods

### Study area

Estonia is situated south of Finland and west from Russia beside the Baltic Sea. The elevation is quite flat, with a maximum height of 317 m over the sea level. While small in size (45 000 km^2^), the variation of climate and geological conditions make the local ecosystems rich in biological diversity (Paal 1998). The geology and soils are complex, with some of the soils situated on Silurian and Ordovician limestones, some on Devonian sandstone, the landscapes have been influenced by the last ice age 11 000 years ago. The climate conditions situate Estonia on the border of the taiga biome of Finland and Russia and deciduous forests of Central Europe. Estonian vegetation consists of coniferous and mixed forests (forest coverage of Estonia is about 50 %), wetlands (bogs and marshes, area coverage initially about 20 %, about quarter of them still relatively intact) and agricultural landscapes (fields and pastures), with some semi-natural habitats (wooded meadows, alvars) (Paal 1998). Estonian plant diversity hotspots are located mostly in Northern and Western Estonia (Kukk *et al.* 2020). However, Southern and Eastern part of the country have slightly distinct flora.

Natural Estonian flora consists of about 1500 species of vascular plants, 50 of them belong to *Pteridophyta* and four to *Gymnosperma* (Kukk 1999). The largest family is *Asteraceae* (354 species), following by *Cyperaceae* (95), *Poaceae* (92) and *Rosaceae* (89). There are ca. 80 widely occurring naturalized plant species and 700 non-native species which sometimes occur in the wild. Thirteen alien vascular plant species have been listed as threatening natural biodiversity (https://www.riigiteataja.ee/akt/12828512). A third of the Estonian natural species have distribution areas in Europe and Siberia, 23 % in Europe and 16 % have circumpolar distribution (Kukk 1999). Estonia is located at the distribution border for one-third of the natural plant species. The most common is NE and N borders (both 8 %), followed by SW (4 %), NW and O (both 3 %), other borders have lower frequencies.

### The *Flora Incognita* application

**Figure 1.**
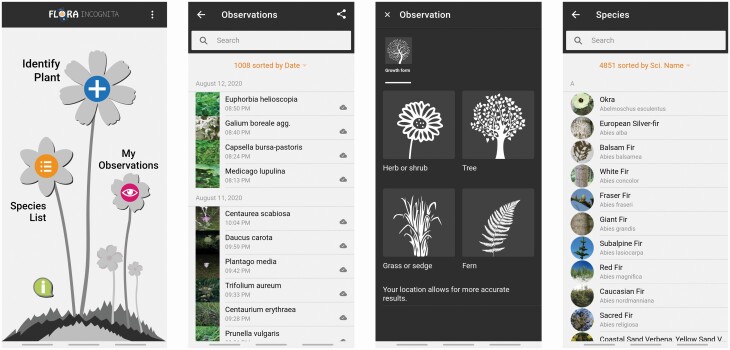
Screenshots of the *Flora Incognita* application (left to right): title page, previous observations page, identification page and the list of species.

We used *Flora Incognita*, a free application developed by the Technical University Ilmenau and the Max Planck Institute of Biogeochemistry (Fig. 1). *Flora Incognita* was originally developed for the German flora. In 2020, the application was already able to identify 4848 vascular plant species covering the Central European flora. Because of this geographic focus, this app seemed to be the most appropriate fort he current study, as the Estonian flora is largely a subset of the Central European flora.

Depending on the difficulty of identification, the application analyses one or several smartphone photos from predefined plant organs and perspectives. Images of the whole plant or of plant organs, such as flowers, leaves or fruits, are gradually transferred to the *Flora Incognita* server until the plant can be identified to species level and the result is then transferred back to the user’s device. Sometimes several taxa are suggested; rarely there are no suggestions when similarity to all species in the application’s database is low. The interactive classifier uses a task-specific CNN cascade, a standard choice for analysing images (LeCun *et al.* 2015). Taxonomy for species is based on Catalogue of Life (CoL), with some complex genera (e.g. *Hieracium*, *Rubus*, *Sorbus*, *Taraxacum*) not fully resolved at the species level. A detailed description of the application can be found at Mäder *et al.* (2021).

### Study settings

We used two different settings. Firstly, we took images from a database and had them identified with the *Flora Incognita* classifier. In the following referred to as the ‘database’ study. Secondly, we tested the application directly in the field. In the following called the ‘field’ study. Table 1 displays an overview of the two study settings.

**Table 1.** Comparison of the database and field study settings.

Factor	Database study	Field study
Identification task	Identification using only the classifier algorithm in a server without the application	Identification with the application directly in the field
Number of species	542	280
Number of observations	1496 (one image each)	998 (with 1703 images)
Number of genera	349	203
Number of families	89	72
Image perspective type	No predefined perspective	Predefined perspectives proposed by the application
Images per identification task	1	Depending on the certainty of identification, *Flora Incognita* required one or more images per identification
Replicates per species	1–16 images (median 2)	1–5 observations (median 4)
Verification of the identification	Record in database (confirmed by an expert)	Dichotomous key book + database expert confirmation
Plant organs: reproductive/reproductive and vegetative/vegetative	496/720/280	332/450/216
Number of species in the image: single species/multiple species	1168/328	776/222
Background: no vegetation/non-natural/vegetation	763/28/705	632/45/321

Combined, our study consisted of 2494 observations with 3199 images from 588 species, 365 genera and 89 families. The selected species were a subset of the 4848 species that can be potentially identified with the application. The database study initially also included images of *Draba incana*, *Lychnis chalcedonica*, *Moehringia lateriflora*, *Rodgersia aesculifolia* and *Salix lapponica*, which are not part of the *Flora Incognita* species list; thus, the machine learning model was not trained to classify them and these species were not included to further analyses. The *Flora Incognita*’s identification results for these species were evidently not successful and typically no species are displayed for the user. For information, we obtained the algorithm’s best matches for these images from the server (**see****Supporting Information—Appendix 1** for more information).

### Details of the database study

We used eBiodiversity—a portal for the taxa found in Estonia (https://elurikkus.ee/en/) in the database study. The eBiodiversity database is developed by University of Tartu Natural Museum and Botanical Garden, it consists of citizen and expert observations and is curated by expert moderators, assuring the quality of the data. The database uses the PlutoF Data management and Publishing Platform (Abarenkov *et al.* 2010).

Field-taken images of native or naturalized vascular plants were downloaded from eBiodiversity. Altogether over 2500 photos were received, the photographs were manually sorted to determine the ones suitable for identification. Most exclusions were images with too low resolution, images of multispecies communities and photographs where the plant individual was out of focus. The selected photos were identified by the same algorithm the application uses but instead of uploading them to the application, the identification took place directly on the project server (service is not publicly available). The identification was conducted in March 2020.

### Details of the field study

**Figure 2.**
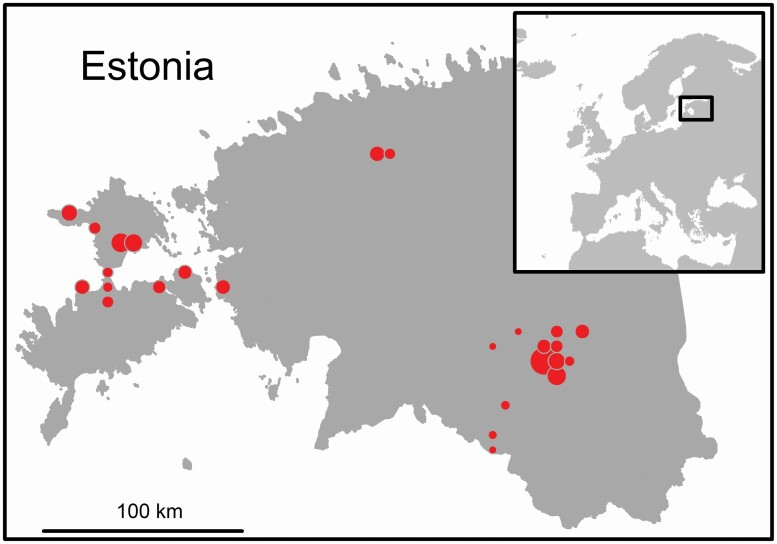
A map of the observation locations of the field study in Estonia (study region marked on the inset map of Europe), the dot size is indicating the number of observations in logarithmic scale (ranging from 1 to 500).

The fieldworks were concentrated to SE and NW Estonia (Fig. 2), as the first has soils on sandstone and the latter on limestone, thus having differences in plant species composition. The observations included plant individuals from varying habitats, including meadows, fields, forests and semi-natural habitats (alvars and wooded meadows). The fieldworks took place from March 2020 until August 2020, therefore including of species with different life cycles and phenological periods, most of the observations were taken in June, July and August 2020.

An observation consisted of: (i) manual identification of the plant individual (using Estonian plant key book; Krall *et al.* 2010). (ii) Automatic identification of the individual with the application. (iii) Inserting the observation into the PlutoF database via Legulus (a data collecting application for PlutoF; https://legulus.tools/#/), the experts of PlutoF database manually verified the traditional identifications. The observations were made with a Samsung Galaxy A40 phone camera. The resolution of the camera was 16.0 megapixels and aperture F/1.7 (Galaxy A40 Enterprise Edition 2021) and it was used with 16:9 ratio and automatic ISO and white balance.

### Data analysis

The taxonomy from the eBiodiversity database, key book identification and that of application’s suggestions were manually unified according to the *Flora Incognita* taxonomy. Synonyms were merged according to Global Biodiversity Information Facility (GBIF.org 2021), World Flora Online (WFO 2021) and Plants of the World Online (POWO 2019). Subspecies and varieties were merged to species level. In some cases the species was used under wide definition, for example *Dactylorhiza baltica* was merged under *Dactylorhiza majalis*, as it can be considered a subspecies of *D. majalis*. World Flora Online taxonomy backbone version 2019.05 (WFO 2021) was used to link the species to the corresponding plant families and to match our data to species’ characteristics in other data sets, we used the R package WorldFlora (Kindt 2020). All observations were divided into four classes: (i) species correctly identified as the first suggestion, (ii) genus correctly identified as the first suggestion, (iii) family correctly identified as the first suggestion and (iv) no correct identification to family level. The identification results from both studies can be found in Zenodo.org repository (see Data Availability section).

We determined the percentages of species in each identification class. A single species often had several observations in both database and field studies. Out of those observations we repeatedly selected a random one. Thus, each species was used once to find the percentages of different identification classes. The selection of one observation per species was iterated 1000 times. Results were averaged from the iterations.

To compare the identification results from database and field study, we only used the species which were present in both data sets (234 in total). Selection of replicates was identical to that of described above. We calculated for each identification class proportion of iterations where the percentage of species from the field study was higher than in the database study. Proportions <0.05 would indicate a significant difference between database and field study in a particular identification class (*P* < 0.05).

In the following analyses we merged the data from both database and field studies. To explore how plant identification varies across taxonomic groups, we selected 16 families (altogether 362 species) with 10 or more species. Percentages of identification accuracy were calculated with iterations as described above. We made a cross table of all combinations and used Fisher exact test in each iteration. Median and maximum *P*-value from 1000 iterations were calculated. We further tested which cell value (combination of identification accuracy class and family) is lower or higher than expected by random. For that we used the averaged occurrences in the table and generated 1000 random tables with given marginals using R stat function r2dtable. This function uses Patefield’s randomization algorithm with fixed row and column sums (Patefield 1981). For each cell we calculated *z*-score as [(observed value − mean random value)/standard deviation of random value]. Significance of *z*-score was obtained from probit function (normal distribution with mean = 0 and SD = 1). Probabilities <0.05 or >0.95 were marked on graphs.

Species frequency was estimated by counting 9 × 11 km grid cells from the Estonian Flora Atlas 2020 (Kukk *et al.* 2020). In each iteration we calculated the median of species frequency in each identification class. In addition, the null hypothesis was created by selecting the same number of species per identification class randomly and calculating median frequency from them. Significances (*P*-values) were found for each identification class as the proportion of iterations where randomized data (null model) gave a larger median than the median from the empirical data.

We compared our results with several characteristics of the species. Plant growth-form data were taken from the *Flora Incognita* application data set, Raunkiær life-form data originated from the BiolFlor database (Kühn *et al.* 2004) and was supplemented with data from the Info Flora database (Info Flora 2021). The main habitat of species was taken from the Estonian flora (Eesti NSV floora 1959–1984). Differences were tested using the Fischer exact test and cell-based randomization, as described above.

All observations from both data sets were annotated according to image characteristics (based on one image in the database study and one or more images from the field study; Table 1). We noted presences and absences of reproductive and vegetative organs of the photographed plant individual (reproductive, both reproductive and vegetative, or vegetative). We marked whether only the pictured species was in focus or other plant species as well. Finally, we classified the background of the vegetation (images where only the pictured plant was in focus, background was either blurred or soil; an artificial background was used, e.g. book cover, photographer’s palm, buildings; or the pictured plant was within other vegetation).

The importance of image characteristics for identification was tested by logistic generalized mixed model (R package lme4, function glmer; Bates *et al.* 2015). We used binomial study variable (identified to species or not) since models with ordered classes did not converge due to very uneven class sizes. In order to filter out the effect of species identity, species was used as a random factor. Data from species with at least three observations were used (2158 observations qualified). We used Analysis of Deviance (type III Wald chi-squared tests, R package car, function Anova; Fox and Weisberg 2019) to find the significance of three annotated image characteristics. We used estimated marginal means to test differences between factor levels (R package and function emmeans; Lenth 2021).

We tested if species-level identification success (proportion of observations identified to species) is related to the number of training images used for *Flora Incognita* (Pearson correlation, the number of training images was ln-transformed). To further interpret our results, we also explored if the number of training images is different among studied plant families, growth forms, life forms and main habitats (dispersal analysis of type III and estimated marginal means *post hoc* comparisons). The correlation between species frequency in Estonia and the number of training images per species was tested with Pearson correlation (the number of training images was ln-transformed).

## Results

**Figure 3.**
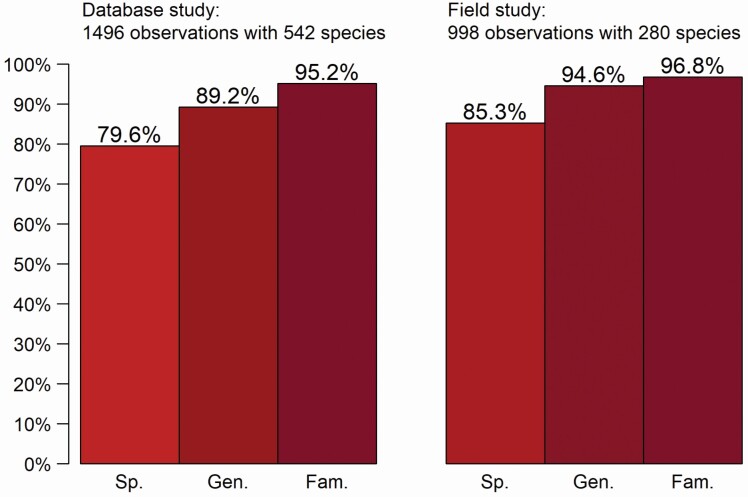
Plant identification accuracy in database and field studies (identification percentages to species, genus and family level).

Both in the database and field study 79–85 % of observations were correctly identified to species (Fig. 3). The plants were identified to at least the correct genus as the first suggestion in over 89 % of the cases. The correct plant family was suggested on more than 95 % of the observations in both studies.

When we compared both studies by identification accuracy only using species common in both data sets, there was no significant difference in the accuracy of database and field identification (*P*-values for different identification classes were between 0.45 and 0.50). In the database study 85.9 % of the species common in both data sets were identified to species level and in the field study the corresponding value was 86.1 %.

**Figure 4.**
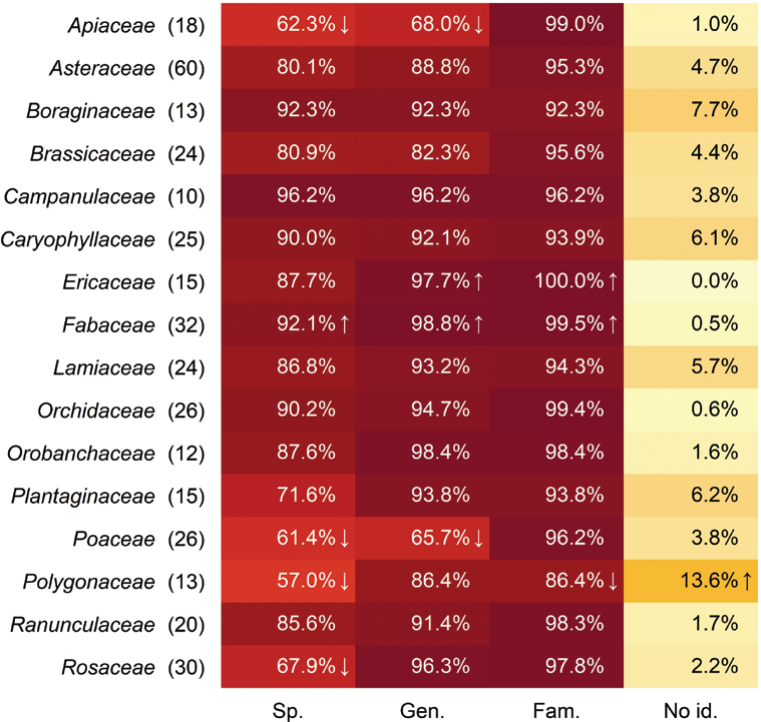
Identification accuracy of species in larger families (number of used species in parentheses). Cells in the table which have significantly larger or smaller values than expected by chance (*P* < 0.05) are marked by arrows up or down, respectively.

Identification accuracy varied greatly among the larger plant families (at least 10 species in the study). Fischer exact test gave a median *P*-value of <0.001, showing a very significant difference between larger plant families in the study (Fig. 4). Compared to random expectation, *Fabaceae* was more often identified at the species level, *Ericaceae* at genus level. Four families—*Apiaceae*, *Poaceae*, *Polygonaceae* and *Rosaceae*—were identified to the species level less than was expected randomly. *Polygonaceae* was more often not identified even to the family level.

There is a total of 540 9 × 11 km grid cells on the Atlas of the Estonian Flora map. The number of cells gives an accurate representation of how frequent the species is in Estonia. We found that the median frequency of species from different identification classes did not differ from the random expectation (*P*-values ranging from 0.112 to 0.475). The main habitat of the species did not make a major difference in identification accuracy, the median *P*-value was 0.22.

**Figure 5.**
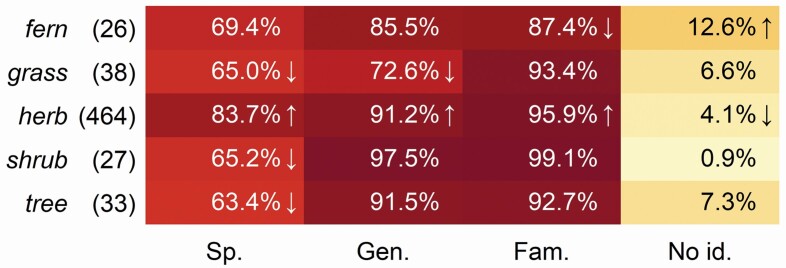
Identification accuracy of species according to the species growth form (number of species in the parentheses). Cells in the table which have significantly larger or smaller values than expected by chance (*P* < 0.05) are marked by arrows up or down, respectively.

The analysis on the growth form of species demonstrated that herbs were identified with 83.7 % accuracy to species level, being a higher value than expected by chance; the other categories remaining between 63.4 and 69.4 % (Fig. 5). Median *P*-value calculated with Fischer exact test was 0.0004, showing a significant difference. Ferns did not reach identification at family level more often than expected by chance.

**Figure 6.**
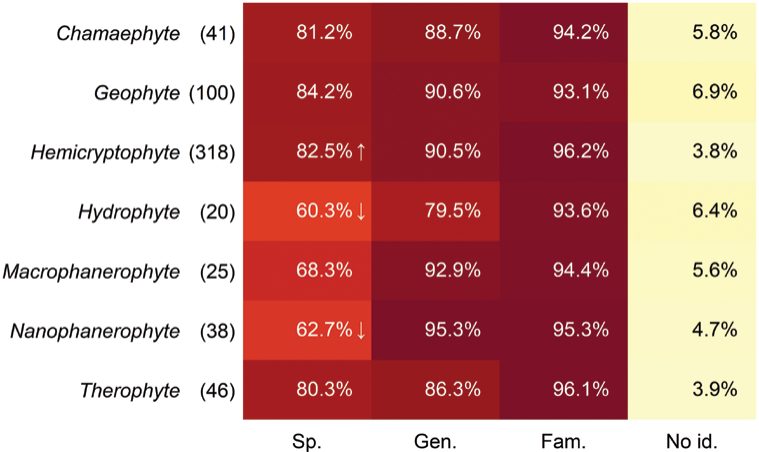
Identification accuracy of species according to the Raunkiær plant life-form categories (number of species in the parentheses). Cells in the table which have significantly larger or smaller values than expected by chance (*P* < 0.05) are marked by arrows up or down, respectively.

The life-form analysis displays that hemicryptophytes were identified at the species level more often than expected (82.5 %) while hydrophytes and nanophanerophytes were identified less often, having values just over 60 % (Fig. 6). Fischer exact test median *P*-value was 0.005.

**Figure 7.**
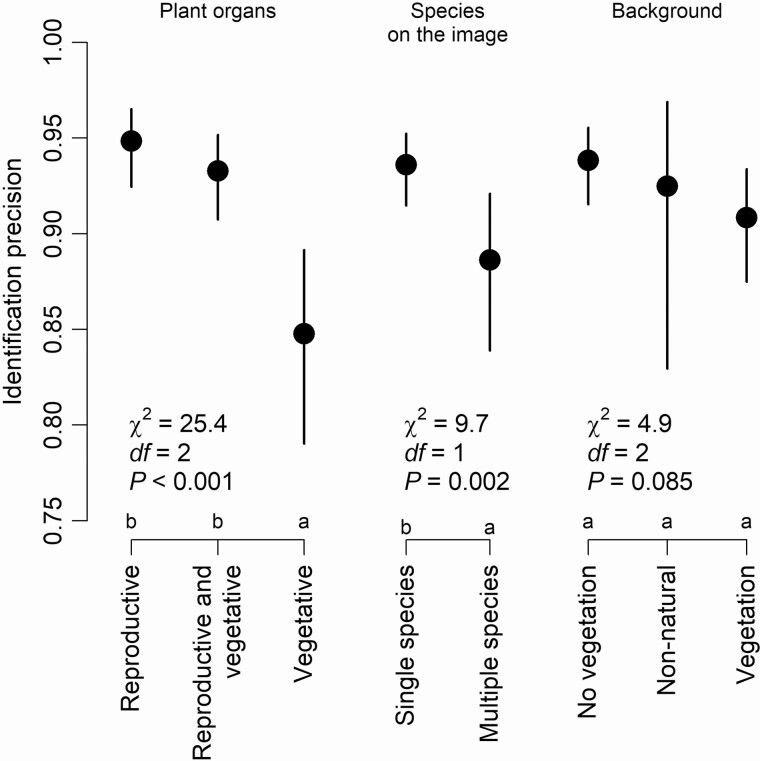
The impact of image characteristics (photographed plant organs, number of species in the image and background) on the identification precision. The dots are displaying expected values when the effect of other variables was into taken account (including the difference between species), and the lines respective standard errors. Model output (chi-square values, degrees of freedom and *P*-values) is shown, and letters differentiate groups which are significantly different according to *post hoc* test (based on estimated marginal means).

When analysing characteristics of observation images while controlling the effect of species, we found that the type of plant organs visible and the number of species in the image affected the identification success (to species level) while image background was not significant (Fig. 7). Images with reproductive organs (with or without vegetative organs) were identified more successfully than images with vegetative organs only. When there is only one species in the image, the image is identified more accurately than one with multiple species visible. **Supporting Information—Appendix 2** presents some examples of these significantly different groups.

**Figure 8.**
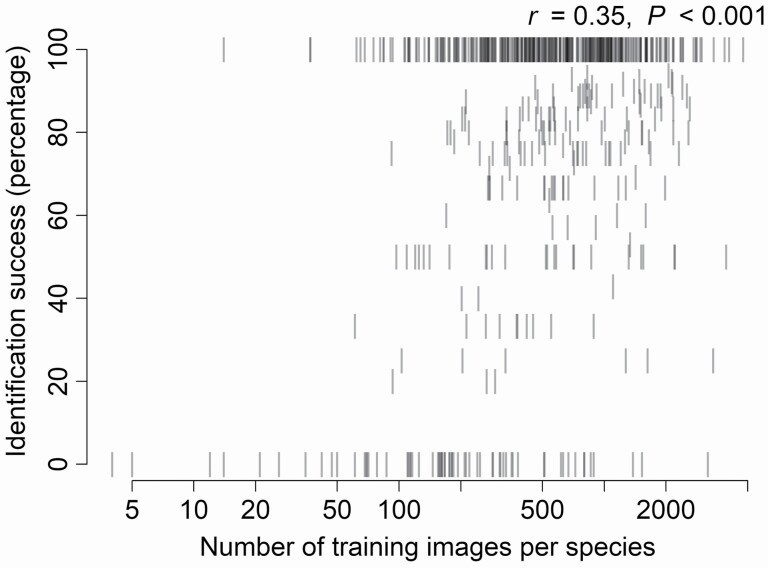
Relationship between identification success of species (percentage of images identified to the species level), and the number of training images used for Flora Incognita machine learning algorithm for particular species. The number of training images is in log-scale on the graph and log-transformation was applied prior to Pearson correlation.

The number of training images per species used for *Flora Incognita* machine learning algorithm was positively correlated to identification success (percentage of images identified to the species level; Fig. 8). The number of training images differed among larger families, growth forms, life forms and species habitats, and it correlated positively with the species frequency in Estonia **[see****Supporting Information—Appendix 3****]**.

## Discussion

With extensive database and field studies we demonstrated that the artificial intelligence-based application *Flora Incognita* was able to identify a great majority of plant images from Northern Europe to the correct species, or at least identify the correct genus or family. The results are comparable to other studies, which, however, included far less species (Schmidt and Steinecke 2019; Biluk *et al.* 2020; Jones 2020; Lüdemann 2020). In field identification, the accuracy of identifying to species was 85.3 %, which outperformed all above-mentioned studies. Although automatic identification already works well, there are still some limitations that need to be overcome in the following years. Our analyses of species and image characteristics that influence the identification accuracy can be used for such improvements.

Contrary to our expectations, the identification was equally good for single images taken from our database and observations taken in the field (often including several images). Moreover, rare and frequent species in Estonia were also identified similarly and the main habitat of the species did not affect the identification accuracy. The lack of difference between database and field study is probably due to the curation of the observations database, which allowed the images to be identifiable by experts. Such an equal performance of the application demonstrates that it could be used widely for several purposes, including identification of threatened plants or general evaluation of biodiversity, which depends largely on relatively common species (Pearmann and Weber 2007). If the quality of the images in a database is reasonable, then such algorithms could also help in identifying the increasing volumes of digitalized plant image collections.

Plant field guides are popular but difficult to use for amateurs (Hawthorne and Lawrence 2006). Identification applications could help bring people closer to plants, as it is common knowledge that key books are useful when the user knows the family of the observable plant, which the application did on 95 % of the cases, meaning that it has the potential to lead the observer on the correct track. Combination of the application and key book could a powerful tool in fieldworks by less experienced observers.

However, our study shows that there was a significant difference in identification accuracy among taxonomic groups, plant growth forms and life forms. Trees and shrubs (macro- and nanophanerophytes) have often been photographed only with leaves for identification. Overall, the same species got a higher identification success when photographed with reproductive organs. Hydrophytes could be identified less accurately because of fewer images in the training data **[see****Supporting Information—Appendix 3****]**. Similarly, automatic plant species identification reaches its limits with species that do not have conspicuous flowers (e.g. *Poaceae* or *Polygonaceae*). The lower performance can either be because the species were poorly photographed due to the fine structure of the flowers or that the typical perspective (e.g. photographing flowers from the top) is not optimal for the identification of the individual. At the same time, there are also difficulties with species that are very similar to each other (e.g. white-flowered *Apiaceae* species). Some progress is expectable, as some initial studies have already been conducted on the ideas how to obtain the most suitable images for automated plant identification (Rzanny *et al.* 2017, 2019).

The main challenge in automated plant species identification arises from the vast number of potential species (Wäldchen *et al.* 2018). In Europe alone (including the Mediterranean basin) there are more than 20 000 vascular plant species. A possible solution is to add geographical information of species’ distribution into the identification process (taken, e.g., from national flora lists or from international databases). Presenting species distributions in key books to support manual species identification has been a common feature. According to initial studies, geographically restricted identification is likely more successful in automated identification (Terry *et al.* 2020). So far, considering metadata like the location or the time of the observation and combining those with the image recognition results has been underused (Wittich *et al.* 2018; Wäldchen and Mäder 2018). However, as traditional key books are regionally specific, geographical restrictions in application should be communicated very clearly to the users. However, a challenge with using geographical information in identification is the dynamic nature of the species distributions, especially amidst global change (Thuiller *et al.* 2008). Species spread actively new regions, and current distribution patterns can be outdated quickly.

In the future, an important development focus should be fine-grained species identification (Šulc and Matas 2017). This requires further development of deep learning technologies and extensive training data sets for these species. Our results confirmed that the number of training images available per species was positively correlated to the identification success. New data collection opportunities through citizen science (e.g. Crocker *et al.* 2020; Boho *et al.* 2020) can broaden the potential sources of labelled image data. Nevertheless, extensive image collection from experienced botanists will play a key role in improving the identification accuracy in the future. The number of training images per species was related to all species characteristics we used (families, growth and life forms, main habitat, frequency). In the future there is a special need for additional training images for most plant families (especially for *Orobanchaceae*), species with low frequency, wetland plants, grasses, hydrophytes and therophytes **[see****Supporting Information—Appendix 3****]**.

In addition to the technical advancements of the identification algorithms, the further development of the application user interfaces plays an important role. Plants in the wild are three-dimensional, and two-dimensional images potentially have limitations to capture all those variations. Images are usually taken in the field with varying external conditions, such as illumination, wind and precipitation. The perception of a human examines the three-dimensional object, not a two-dimensional snapshot of the plant (Wäldchen *et al.* 2018).

It is very important to provide the users with precise guidelines on how the pictures should be taken. The users must be made aware that the image quality and certain plant perspectives are essential for a reliable automated identification. There are initial studies investigating which plant perspectives are important for identification (Rzanny *et al.* 2019). However, these must be expanded for further species groups. We found that images with a single plant species in focus obtained a higher identification accuracy compared to images when several species were present. Occasionally, the application identified another species than was intended by the observer. Plant background, however, was not affecting the results. Thus, when using plant image identification applications, it is recommended to prefer reproductive organs and keep just a single species in focus. It would be desirable if algorithms would inform the user directly in the field that the corresponding image does not meet the quality standards and ask the user to repeat the image acquisition.

The *Flora Incognita* application already gives the user precise instructions on which perspectives of the plants should be photographed. The choice of the perspective depends on different growth forms, which are previously selected by the user. With the ever-improving camera technology in smartphones, even better pictures will be possible in the future and will thus also increase identification rate. However, for future development of the applications, it should be considered to couple the automatic identification with a manual one. This means that in case of an uncertain automatic identification, a multicriteria key should be used to manually query characteristics of the potential species. The application sometimes suggests alternative species, and if we include those, the correct species is mentioned in over 90 % of the cases (results not shown). We are confident that the combination of traditional and automated identification will be a promising avenue.

Plant identification applications have recently made rapid progress and are already usable for several purposes, especially if their capabilities and limitations are known. In particular, they can improve the situation concerning plant blindness and also foster citizen science. With the number of observations with photographs growing, high-quality training data for machine learning algorithms are increasing in number; therefore, the identification applications have the potential for even more accurate results in the near future. Our results can be used to improve *Flora Incognita* and other plant identification applications, for example, putting extra effort to increase training images for specific species groups, providing better guidelines for application users for photographing, or asking for additional information for specific taxa.

## Supporting Information

The following additional information is available in the online version of this article—

Appendix 1. Results for the species not in database.Appendix 2. Examples of image characteristics affecting the identification results.Appendix 3. Analyses of training data per species used by *Flora Incognita*.

plab050_suppl_Supplementary_Materials

## Data Availability

The data set used in the analysis is available in the Zenodo.org repository (https://doi.org/10.5281/zenodo.4761451). The data set includes all of the images used in the observations, 3199 in total, and a data table with all of the observations and their traits (all of the data from the analysis for the species/observation are included).

## Notes

**Plants, Ecosystems & Climate. Chief Editor:** Mary Heskel
