# Supplementary material for: Plant image identification application demonstrates high accuracy in Northern Europe
Source: AoB Plants. 2021 Jul 27;13(4):plab050. doi: 10.1093/aobpla/plab050 (PMC8387968; doi:10.1093/aobpla/plab050)
Supplement: plab050_suppl_Supplementary_Materials [file plab050_suppl_supplementary_materials.pdf]

Supporting information for:

Pärtel, J., Pärtel, M. & Wäldchen, J. 2021. Plant image identification application demonstrates high accuracy in Northern Europe. AoB PLANTS.

Appendix 1. Results for the species not in database

Taxa not known to the machine learning model, i.e. those on which the classifier was not trained, could not be recognized. The model is unable to determine whether an image belongs to an unknown taxon it has never seen before and will wrongly identify a known taxon with the highest degree of similarity. Typically, the highest degree of similarity is still so low that no suggestions will be given for the user. Here we extracted the best match information directly from the *Flora Incognita* server but did not use these species in our analyses since the model's prediction can only be interpreted in a meaningful way for taxa on which it was actually trained. The five unknown species were only found in the database study. Some of them were garden plants, or had a northern or eastern distribution. (1) *Draba incana* has an arcotmontane distribution which reach its SW border in Estonia, the most similar species in the *Flora Incognita* database was *Arabis hirsuta*. (2) *Lychnis chalcedonica* originates from continental Eurasia but is used as an ornamental garden plant. In Estonia it sometimes spreads to nature. *Flora Incognita* is focusing on European natural plants, even if many naturalized ones are included. The most similar species found was *Phlox paniculata*. (3) *Moehringia lateriflora* is distributed in Eurasia, reaching in its E border in Estonia. The most similar match was *Cardamine californica.* (4) *Rodgersia aesculifolia* originates from China but is used as an ornamental plant in Estonia, occasionally found in nature close to settlements. The best match from the *Flora Incognita* database was *Aesculus hippocastanum* due to the similarity of leaves. (5) *Salix lapponum* is distributed in Northern Eurasia, *Flora Incognita* identified Equisetum *arvense,* which was actually in the image as well.

Appendix 2. Examples of image characteristics affecting the identification results

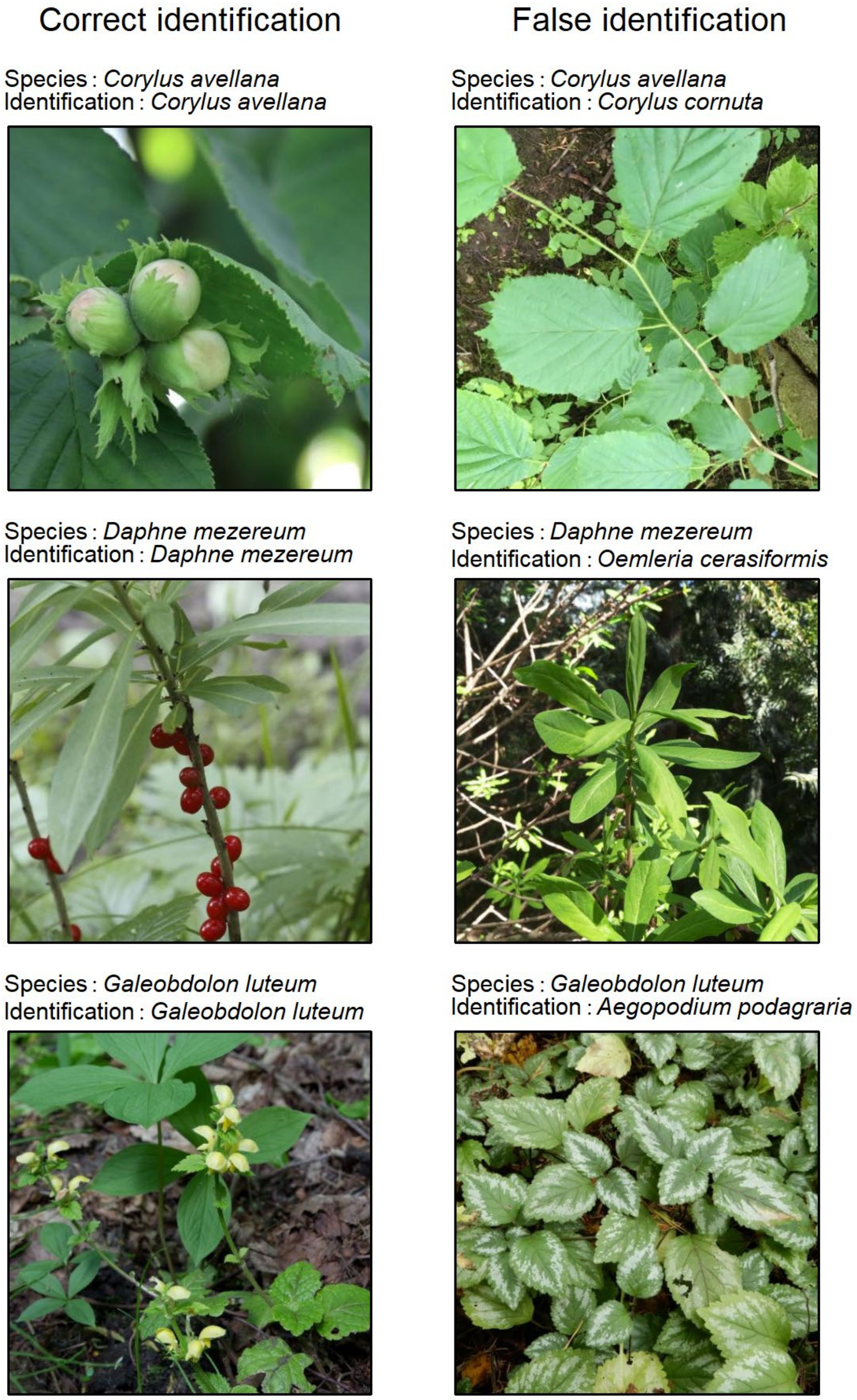

Figure S2.1. Correct and false identification of species with and without reproductive organs

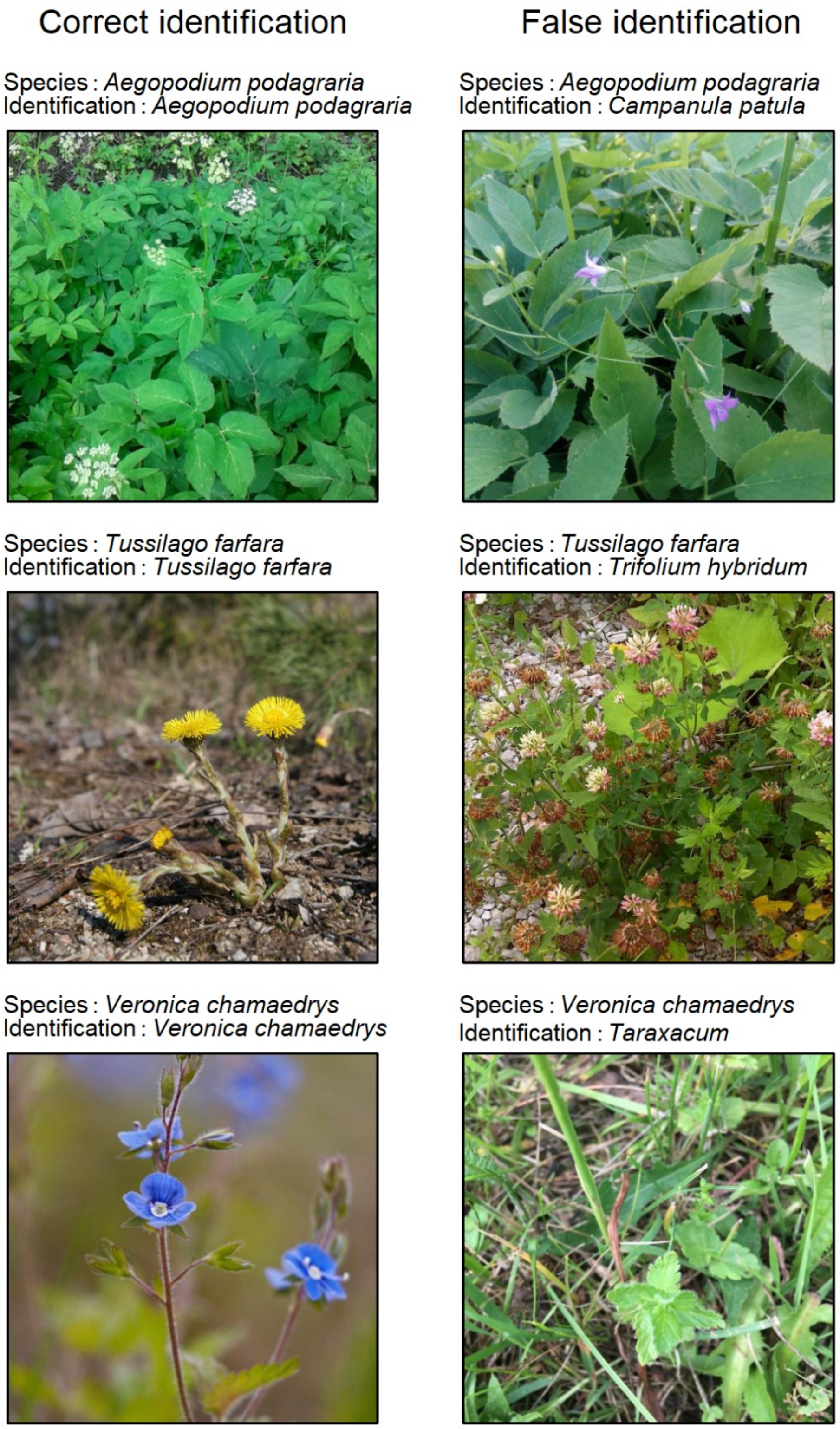

Figure S2.2. Correct and false identification of images where the target species is alone on the image compared to images with multiple species (application identified another species than the photographed one)

Appendix 3. Analyses of training data per species used by *Flora Incognita*. Relationships with parameters used in the current study

The number of training images varied greatly across the species. Minimum number of training images was 4 (*Silene chlorantha*), maximum 4754 (*Taraxacum sp.*), median was 540.5 and mean 745.4 images per species.

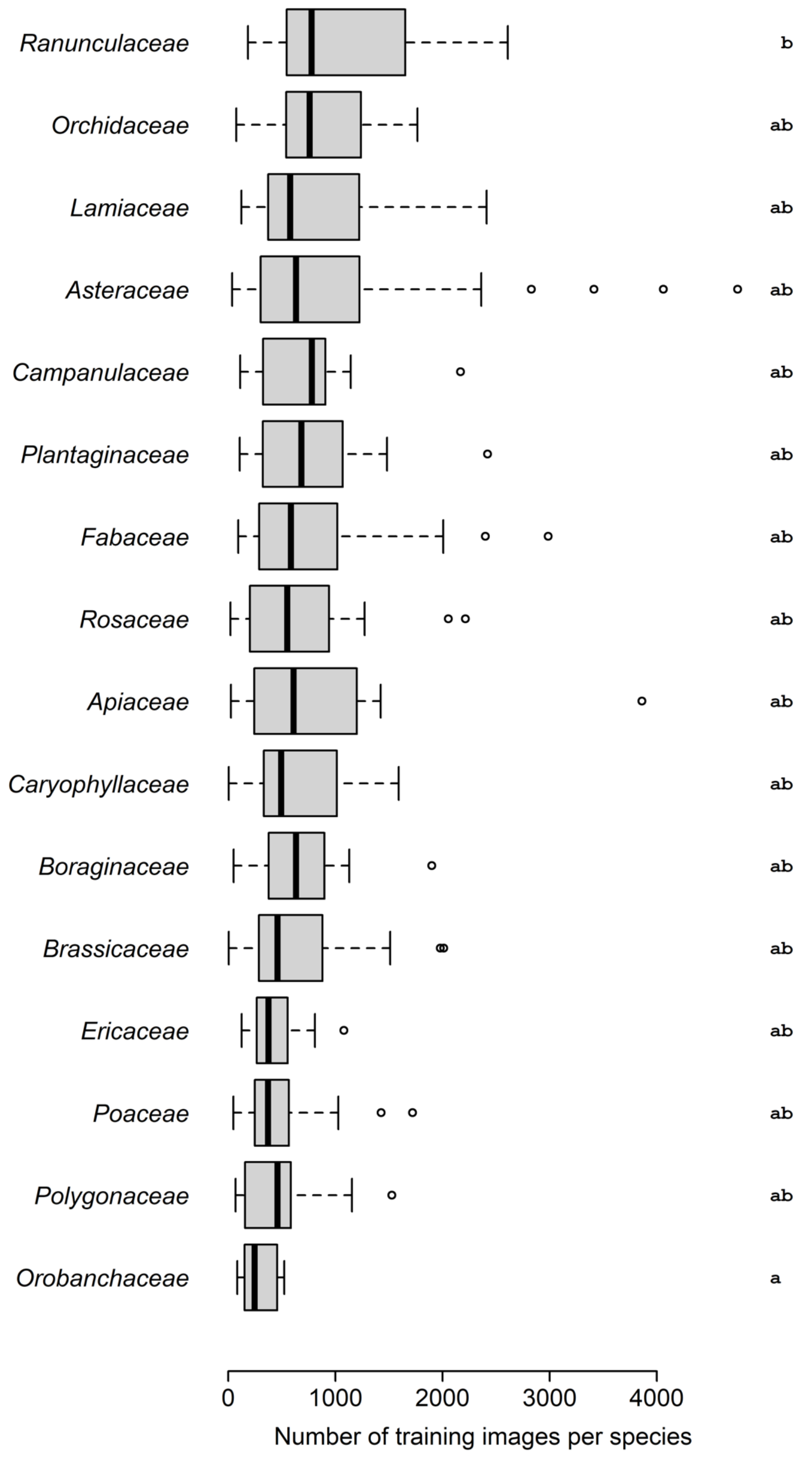

Figure S3.1. Numbers of training images per species in larger plant families, $F_{15,347} = 1.8$, $P = 0.028$. Groups with same letters does not differ from each other.

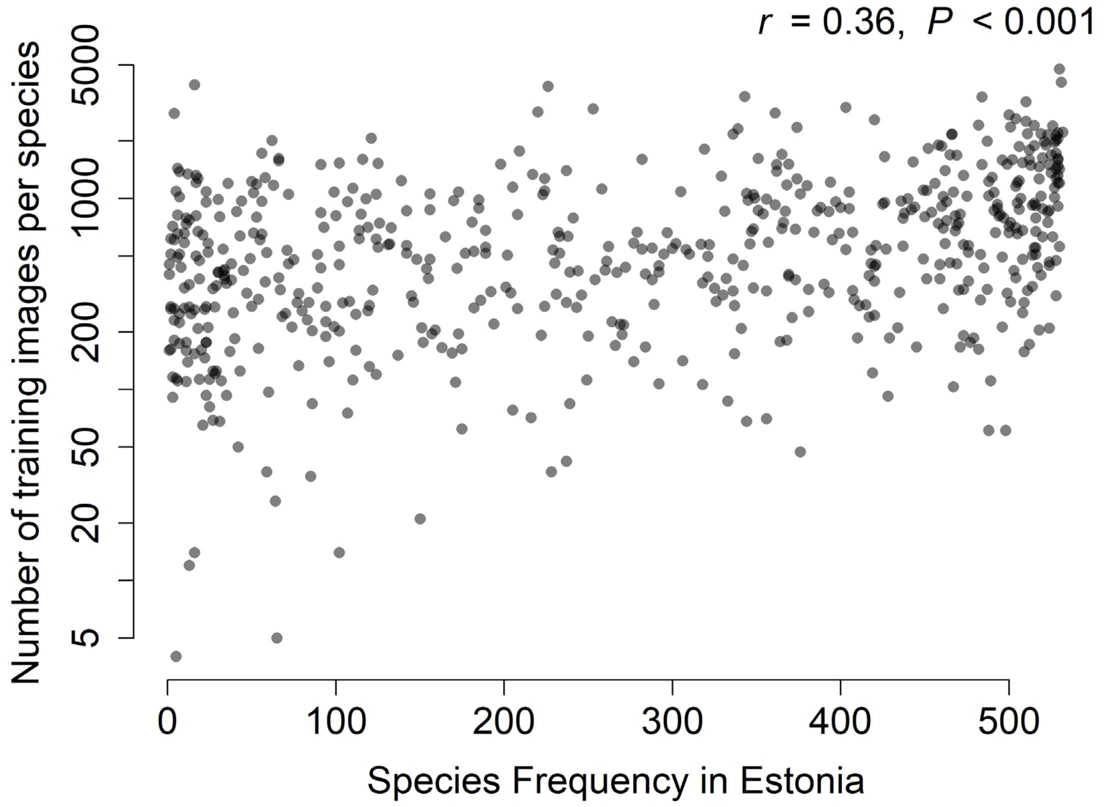

Figure S3.2. Species' frequency in Estonia is correlated with the number of training images available for *Flora Incognita*. The number of training images is in log-scale and log-transformed for Pearson correlation test.

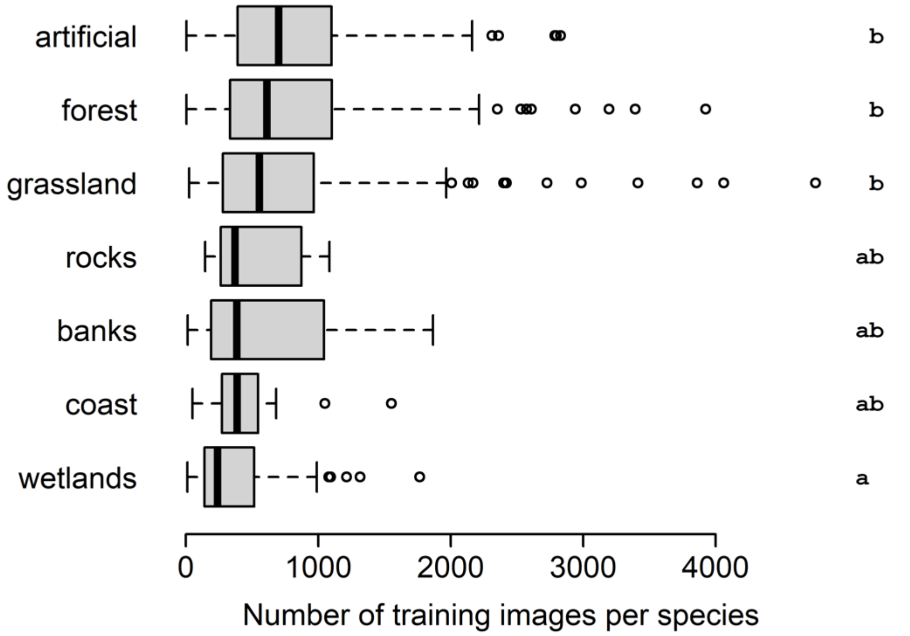

Figure S3.3. The number of training images per species across the main habitat of the species in Estonia. Artificial habitats include gardens, fields, urban areas and wastelands, wetlands include also water plants. $F_{6,581} = 7.7$, $P < 0.001$, Groups with same letters does not differ from each other

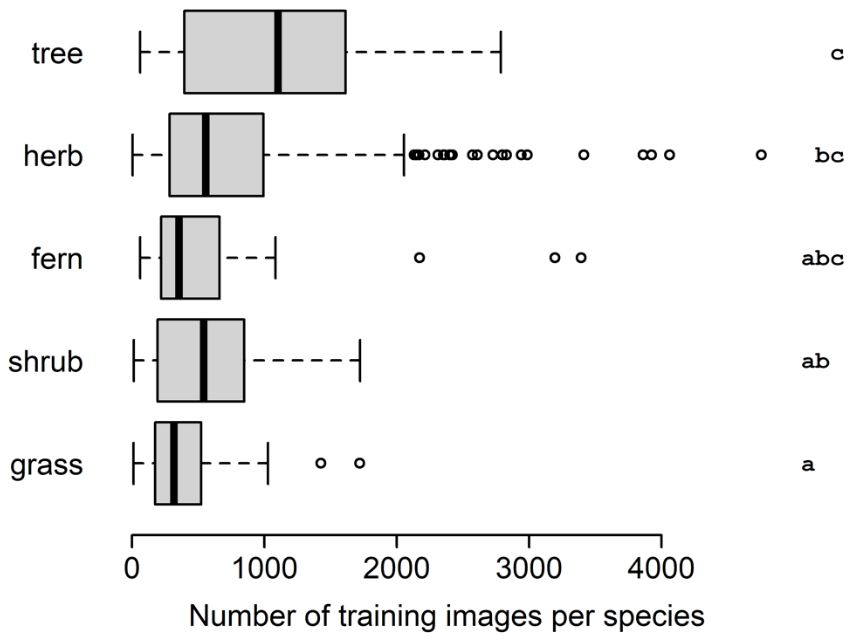

Figure S3.4. The number of training images per species across plant growth forms, $F_{4,583} = 6.8$, $P < 0.001$. Groups with same letters does not differ from each other

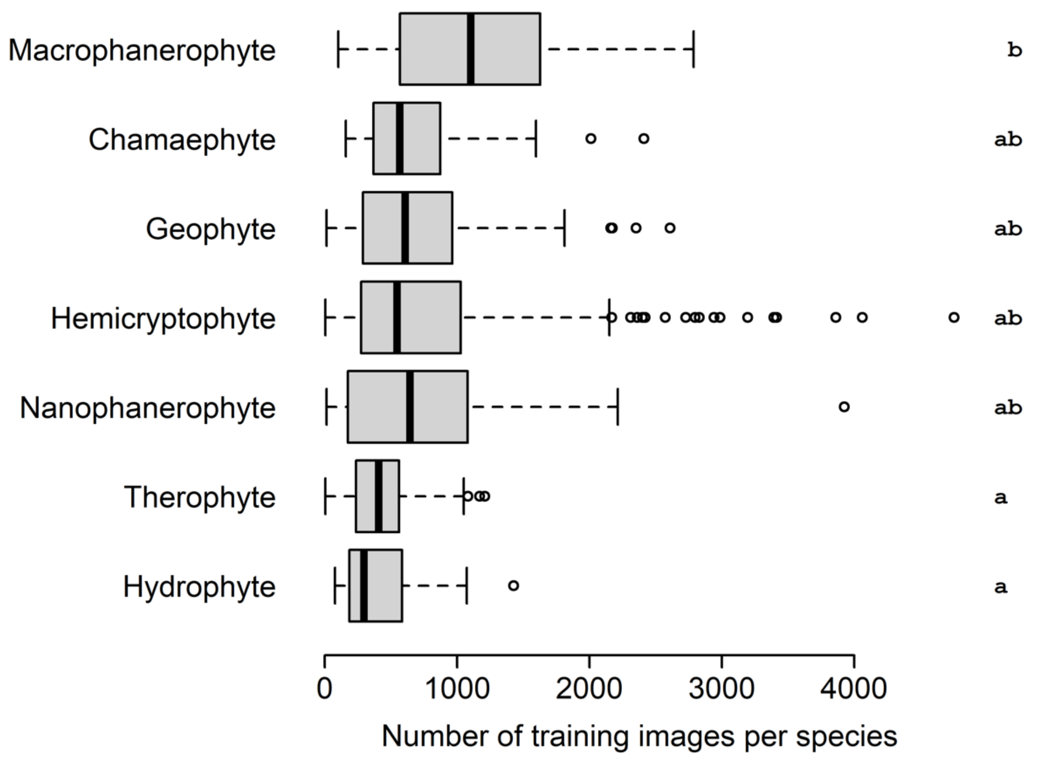

Figure S3.5. Number of training images per species over the life-form of the species, $F_{6,581} = 3.6$, $P = 0.001$. Groups with same letters does not differ from each other
